# Pharmacists’ perspectives on recommending herbal medicines for acute infections: a qualitative study

**DOI:** 10.3399/BJGPO.2023.0138

**Published:** 2024-03-20

**Authors:** Xiao-Yang Hu, Martin Logue, Emma Maund, Miriam Santer, Merlin Luke Willcox, Shazab Islam, Tom Stokes, Michael Moore

**Affiliations:** 1 Primary Care Research Centre, School of Primary Care, Population Sciences, and Medical Education, Faculty of Medicine, University of Southampton, Southampton, UK; 2 Southampton Health Technology Assessments Centre, School of Healthcare Enterprise and Innovation, University of Southampton, Southampton, UK; 3 University Hospitals of North Midlands NHS Trust, UK; 4 Public contributor, Southampton, UK

**Keywords:** pharmacists, self-management, qualitative research, herbal medicine, patient preference, antimicrobial stewardship, respiratory tract infections, urinary tract infections

## Abstract

**Background:**

Community pharmacists have an essential role in antimicrobial stewardship by providing self-care advice for self-limiting infections.

**Aim:**

To explore community pharmacists’ perceptions and experiences of advising patients on management of acute respiratory tract infections (RTIs) and urinary tract infections (UTIs), and to explore issues regarding use of over-the-counter (OTC) medicines, including herbal medicines.

**Design & setting:**

A qualitative study using semi-structured interviews with community pharmacists in England.

**Method:**

Qualitative interviews with community pharmacists were carried out face to face and by telephone between November 2019 and March 2020. Data were collected through in-depth, semi-structured interviews, recorded and transcribed. A reflexive thematic analysis was undertaken.

**Results:**

In total, 18 community pharmacists were interviewed. Three main themes were identified. Theme 1 was self-management recommendations. Community pharmacists considered patients’ preferences when recommending self-management strategies. Some believed that conventional OTC medications had quicker and stronger effects, while others preferred herbal OTCs as a more natural approach, particularly for less severe symptoms. Theme 2 was factors influencing pharmacists’ recommendations for acute infections. This included pharmacists' perceptions of patient preferences, nature or severity of illness, research evidence, training, commercial pressures, and patient concerns about medication cost. Theme 3 was pharmacist–patient communication. Pharmacists sometimes experienced challenges with language barriers and patients’ expectations of receiving antibiotics. Pharmacists emphasised the importance of being trusted by their patients. There was widespread acceptance of their role in self-management advice for acute illness and interest in the role of herbal medicines, but pharmacists did not feel confident in recommending these.

**Conclusion:**

Pharmacists are central to the management of self-limiting infections. There is a need to educate the public about appropriate use of antibiotics and provide training and support for pharmacists on self-management strategies including herbal medicine.

## How this fits in

Community pharmacists play a critical role in providing self-management advice for acute conditions, which is expected to expand owing to current policy directives. Over-the-counter (OTC) medicines, including herbal medicines, are a useful approach to symptom management, but pharmacists generally lack the confidence to provide advice in this area owing to a perceived lack of evidence. Although some herbal medicines are recommended in clinical guidelines, pharmacists require additional training to confidently incorporate them into their practice. Pharmacists may experience a conflict of interest between their business imperative to sell products, and their public health role to provide evidence-based advice on the most cost-effective medications.

## Introduction

Community pharmacies are central to the strategic vision of healthcare delivery in England.^1^ The Community Pharmacy Contractual Framework incentivises community pharmacies to expand and improve their range of clinical services, setting out plans for urgent care, prevention, and medicines optimisation.^2^ To alleviate pressure on GP appointments, the NHS GP Community Pharmacist Consultation Service was launched in October 2019, to take direct referrals to community pharmacies from both NHS 111 and GP teams for patients presenting with minor illnesses.^3^ It is estimated that 5.5% of all GP consultations (>18 million appointments per year) and 3% of accident and emergency consultations for common ailments (>0.65 million consultations per year) could be safely managed in community pharmacies, which would potentially save the NHS >£1 billion a year.^4^

Antimicrobial resistance (AMR) is an ever-increasing threat to global public health, largely driven by overuse of antibiotics.^5^ In England, general practice accounts for 84% of antibiotic prescribing.^6^ Improved self-care could potentially reduce consultations and unnecessary antibiotic use. Patients’ behaviour is a key target of the 2019–2024 UK AMR national action plan.^7^ Supporting people to self-manage self-limiting infectious conditions is recommended as a first-line strategy by the National Institute for Health and Care Excellence (NICE)^8^ and is consistent with the NHS Long Term Plan to provide personalised care.^9^ In providing patients with effective self-care treatment advice^10^ and recommending OTC treatment for common infectious conditions,^11^ community pharmacists play an essential role in antimicrobial stewardship.^12^ The number of medications available OTC without a prescription is rapidly increasing^13^ and prescription medicines including antibiotics can be offered using patient group directives (PGDs). The introduction of the Pharmacy First service in early 2024 marks a notable stride towards pharmacists becoming independent prescribers. This service, initiated in response to the growing demand for accessible health care, not only expands the role of pharmacists but also underscores their increasing autonomy in prescribing medications.

Herbal and traditional medicines, such as *Pelargonium sidoides* ^14^ and *Andrographis paniculata,* ^15^ may be promising safe and effective alternatives to aid in alleviating symptoms of acute respiratory infections (ARIs) and cough.^16^ NICE suggest self-management treatment, such as honey and pelargonium, for the relief of cough, and recommend cough medicines containing the expectorant guaifenesin or cough suppressants, except codeine, for relieving acute cough symptoms.^8^ NICE also recommend D-mannose and cranberry products to reduce the risk of urinary tract infections (UTIs).^8^ In the UK, herbal products, such as Kaloba and AsParAid, are registered under the traditional herbal registration (THR) scheme to ensure safety, quality, and regulatory compliance of herbal products. However, numerous herbal products are available on the market without a registration and the responsibility falls on manufacturers and distributors to ensure their products meet quality and safety standards. Previous qualitative studies have explored pharmacists’ perspectives on self-management of long-term^17^ sleeping problems in older people^18^ and stress,^19^ but none,^20^ to the authors’ knowledge, have investigated what factors influence their advice on self-management for acute infections.

Given these important policy directives and planned changes in service delivery, this qualitative research aimed to explore the perceptions and experiences of community pharmacists around giving advice to patients on management of acute respiratory tract infections (RTIs) and UTIs, and to explore specific issues regarding the use of herbal medicines as part of their self-care plans.

## Method

This qualitative study is reported following the Standards for Reporting Qualitative research (SRQR) checklist.

### Recruitment

The recruitment took place between November 2019 and March 2020. A variety of methods were used to advertise the study to potential participants. Email invitations were sent to members of the following groups/networks:

- local pharmacy practice committees via pharmacy leads;
- National Institute for Health and Care Research clinical research networks; and
- Community Pharmacy South Central (https://cpsc.org.uk), a committee representative of the pharmacy contractors in the areas of Health and Wellbeing Boards for Southampton City, Hampshire, Isle of Wight, and Portsmouth City.

Community pharmacists were also identified through searching online for pharmacies in London and Oxford, phoning them directly, and opportunistically by visiting the pharmacies.

### Sampling

Participants’ demographics, including sex, age, and the number of years they had worked in pharmacies, type of pharmacy, and pharmacy postcode (to calculate index of multiple deprivation), were collected to inform sampling and reporting. Deprivation index was checked against the English indices of deprivation 2015.^21^ A purposive sample with a maximum variation approach^22^ was used to identify participants and to obtain a broad range of perspectives from participants in London, Oxford, and the Wessex region, which included sex, work experience, type of pharmacy (large, small, or independent pharmacies), and deprivation index.

### Data collection

Qualitative semi-structured interviews were conducted to provide in-depth findings regarding community pharmacists’ experiences and views on giving advice to patients on management of acute RTIs and UTIs. Interviews were carried out face to face or by telephone by a researcher (ML) with training in qualitative research and herbal medicine. The research team includes expertise in herbal medicine research (ML, XYH, and MLW), primary care research (MS, MLW, and MM), qualitative methodology (XYH, ML, EM, MS, and MLW), and patient and public involvement (TS). The interview guide (see Supplementary Information S1) was informed by existing literature, further developed with input from the wider research team, including public contributors, and piloted with a former community pharmacist (SI) who worked as part of the research team.

Data from earlier interviews were used iteratively to refine the interview guide and revise wording of questions. Informed consent was obtained before the interviews. Interviews were audio-recorded and transcribed verbatim by a professional transcriber.

### Information power

We aimed to recruit a sample of 15–20 community pharmacists, which we considered would provide sufficient depth and richness of data for analysis to answer our research questions concerning different types of pharmacies and pharmacists based in areas with varied deprivation. Data collection continued until no more important novel responses were received taking information power into consideration.^23^ Both negative and positive comments regarding their views on using herbal medicines were encouraged.

### Data analysis

A reflexive thematic analysis approach following the steps of Braun and Clarke was taken,^24^ where the researcher’s active role in knowledge production is recognised.^25^ All transcripts were coded line-by-line with initial codes (ML and EM). Transcripts were compared within and between each other, aiding the iterative search for themes, which were then reviewed, defined, and named. Themes were produced by organising codes around a central organising concept, interpreted by the researcher from the data.^25^ Divergent views were searched and compared. Three transcripts coded by three researchers (ML, XYH, and MLW) to generate initial codes and ensure that multiple perspectives informed the analysis. NVivo (version 12) and Microsoft Excel (2019) were used to facilitate management of the dataset.

### Patient and public involvement

Two public contributors (Margaret Bell and TS) provided input on the aim and design of the study, particularly the method of recruitment, the interview topic guide, and the plain English summary. TS was involved in developing codes and themes during data analysis and in developing outputs from the study.

## Results

Out of 80 community pharmacists contacted, 20 responded and 18 consented to participate, with one in-person and 17 via phone. The interviews took place between November 2019 and March 2020 and lasted from 18–69 minutes (average 42 minutes). Participants’ characteristics are presented in Table 1. Pseudonyms have been given for participants in order to protect identities.

**Table 1.** Characteristics of the study population

Pharmacist characteristics	*n*
**Sex**	
Female	10
Male	8
**Age, years, median (range)**	40 (26–68)
**Ethnic group**	
Asian or Asian British	10
White, British, Greek, or Croatian	7
Black British Nigerian	1
**Years in pharmacy, median (range)**	18 (3–45)
**Pharmacy type** ^**a**^	
Chain	11
Independent	8
**Pharmacy IMD,** ^**b**^ **median (range)**	7 (2–9)

^a^One pharmacist worked part-time in both an independent pharmacy and in a chain pharmacy. ^b^Decile 10 represents the least deprived 10% of neighbourhoods in England. IMD = Index of Multiple Deprivation.

**Figure 1.**
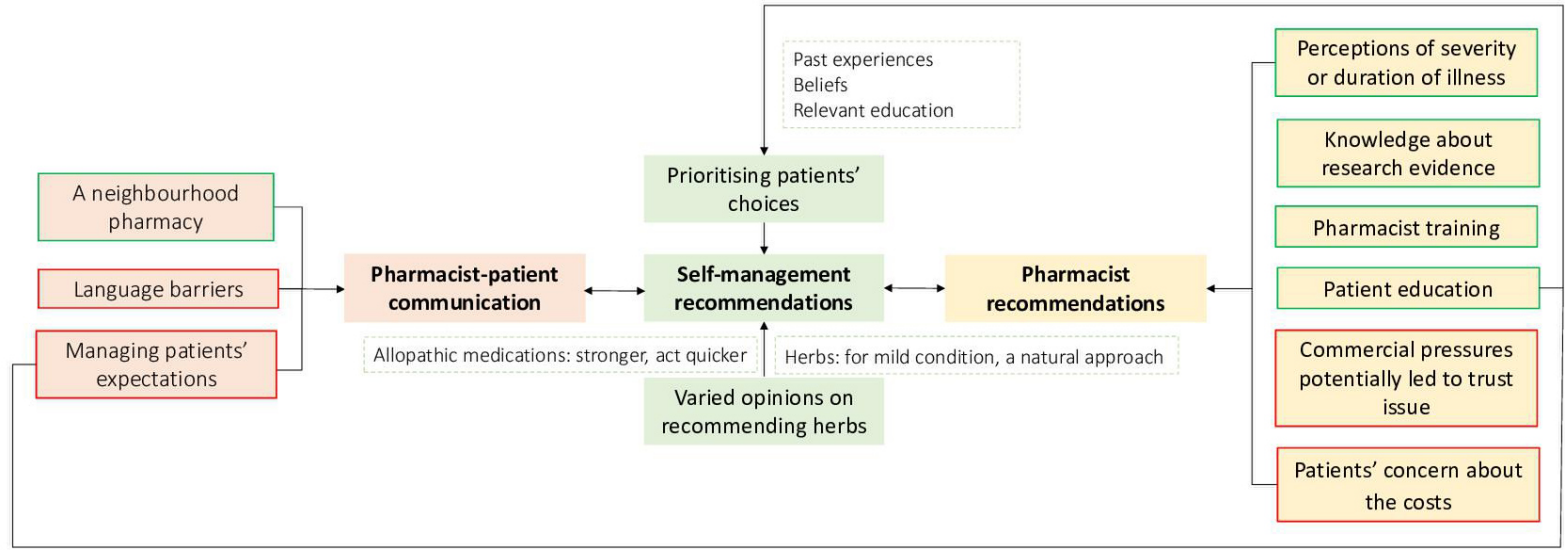
Themes and sub-themes identified from the data. Colour of border indicates link to the respective theme.

The following three themes were identified from the data (Figure 1): self-management recommendations; factors influencing pharmacists’ recommendations for acute infections; and pharmacist–patient communication.

### Theme 1: Self-management recommendations

Pharmacists spoke about their role in providing self-management advice for acute conditions. In particular, the various self-care recommendations that they gave based on patients’ symptom type and severity. These included general lifestyle advice such as drinking plenty of water, steam inhalation, conventional OTC products, and herbal products.

#### Varied opinions on recommending herbs

Pharmacists had varied responses regarding self-management advice for self-limiting conditions. When asked about herbal medicines, some preferred conventional non-antibiotics owing to perceived scientific explanations, while others recommended herbs as a first option:

*'In terms of recommending, like I said, I would still first go with something that’s drug related; of course not like antibiotics, but something that I know that’s proven that’s worked, something that has a mechanism.'* (Jane, 3 years‘ experience, chain)

*'If it’s been within one or two days, then I would always go with herbal because I myself personally don't like going with chemicals/medicinal stuff straight away, so I would always do that.'* (Monica, 14 years‘ experience, chain and independent)

For those who recommended using herbal OTCs, there was a perception that conventional medications act quicker and are stronger, while herbs are for patients with less severe symptoms, as a more natural approach:

*'We do keep a range of them* [cough medicines] *and essentially most of them do have the same ingredients, but of course, first I'd ask them if they want something herbal or something stronger which has a drug in it … They can get a free prescription from the doctor that is going to work quicker* [than herbs]*.'* (Jane, 3 years’ experience, chain)

When sharing experiences, without the interviewer asking about a specific herb, pharmacists often lumped herbal products together. Some believed many popular herbal OTCs for cough have *'essentially got the same ingredients'* (Jane, 3 years’ experience, chain; Juliet, 45 years’ experience, chain) or recommend a product because *'it was such a well-known brand'* (Anna, 35 years’ experience, chain).

#### Prioritising patients’ choices

Pharmacists talked about how they tailored advice according to individual circumstances; for instance, what advice they gave for busy people versus what they would give to other patients:

*'”Listen, I can't be carrying around a bottle. I'm a businessperson. I work in the city.” That’s fine, you have a few lozenges. Sucking some lozenges, you're producing more saliva, coats the throat, makes you feel better, that’s it. Drink lots of fluids, increase your vitamin C consumption. Yes, the usual healthy living things, and it can work.'* (Tom, 16 years’ experience, chain)

Pharmacists also described how patients’ preference or choices commonly appeared to be influenced by their past experiences, beliefs, and relevant education. They thought it was important to present the options to patients and let them make their own choice:

*'I like to give people a choice, I think it’s important. If people feel it’s going to make them better and they have confidence and it’s what they like to choose, I'm happy with that. It needs to be their choice, and them given the options. The proof will be in how they feel and how they respond, they just won't, perhaps, try it again, if it doesn't work for them.'* (Sonia, 35 years’ experience, independent)

### Theme 2: Factors influencing pharmacists’ recommendations for acute infections

Pharmacists shared various factors influencing their self-care recommendations, encompassing the perception of severity or duration of the illness, the presence of research evidence for different therapeutic approaches, their own training, and the provision of educational programmes for patients, commercial imperatives, and patients’ preference, which included concerns regarding the cost of OTC medications.

#### Perceptions of severity or duration of illness

Community pharmacists shared their decision-making process when they saw patients with acute RTIs or UTIs, particularly regarding whether to offer them self-management recommendations or refer them to the GP. They described these decisions as depending on the patient’s age and the pharmacist’s perception of the nature of their symptoms, with a particular focus on the severity and duration of symptoms:

*'We also have to assess the red flags in every situation, so obviously if it’s a new persistent cough that someone over the age of 50 or 55 has got, and it’s been ongoing. Say, something longer than three weeks to four weeks, then anything that falls under a red flag, then we wouldn't treat that, we would refer that straight to a doctor … '* (Andrew, 14 years’ experience, independent)

RTIs or UTIs were not always differentiated, although there was more discussion around using herbal OTCs for treating RTIs and as a preventive measure for UTIs, while many patients expected to get antibiotics for their UTIs:

*'Your antiseptic for the urinary tract, if you can get them quite often you can consider maybe taking* [herbal medicine]*, a couple of days before sex and after a few days and then see if that actually helps … In respect to UTIs because the older there are, generally, they tend to expect antibiotics much more in my scenario.'* (Harry, 16 years’ experience, chain)

#### Knowledge about research evidence

Pharmacists acknowledged the importance of effectiveness of treatments being guided by research evidence and clinical guidelines. Some expressed concern that there was insufficient good research on some products for acute infections, including herbal products:

*'I know there’s not sufficient research in the cough mixtures, or there is research, but there’s no evidence to say that they actually do any good at all, and that’s been the case for many years.'* (Stephen, 30 years’ experience, independent)

There was discussion around how pharmacists research products themselves before stocking and recommending herbal medicines:

*'Unless I do my own research then yes. My colleague who is actually a pharmacist and the owner of the pharmacy where I work, she’s trained a lot in herbal. She’s done a separate course as well on herbal so she normally does that reading and looking into the products on what to stock and what to keep. I wouldn't recommend it unless I had seen it* [the evidence] *myself.'* (Jane, 3 years’ experience, chain)

#### Pharmacist training

When questioned about advising patients on herbal medicines, a major talking point was around evidence and training of pharmacists, particularly in the context of self-management for acute infections and antibiotic reduction. While most expressed desire to enhance their knowledge of non-antibiotic medications, including herbal medicines, options varied on the preferred training format. Some preferred online training for convenience and flexibility, while others believed in-person training would be more valuable:

*'I find that, having gone on certain types of training and undertaken online training and multiple-choice questions, physically reading something and doing workshops face-to-face, as a group – The interaction in a group and face-to-face learning, I find I take it on a lot more and far more interesting. You get to hear from other people. You get to hear their stories and their take on things.'* (Molly, 8 years’ experience, independent)

#### Patient education

Some pharmacists talked about their role in educating the public around self-management of minor illnesses and providing a free information service to ease the load on the health service:

*'We need to educate patients not to ask for things that they don't need, and we need to have some sort of service where they can get things for minor illnesses without having to pay. That would therefore free up the doctors' time and save the health service money. It’s quite complicated! It’s not just a simple fix!'* (Juliet, 45 years’ experience, chain)

They also talked about the need to educate the public specifically about antibiotics:

*'The thing is by promoting campaigns in pharmacies and even in the media, social media, whatever, that antibiotics are not, by using antibiotics on a frequent basis you're going to get resistance, and obviously over the years they're obviously not going to work and when you do need it it’s going to be non-effective.'* (Stephen, 30 years’ experience, independent)

#### Commercial pressures influence recommendations

There was frequent discussion about the tension between working as a health professional and the commercial imperatives to sell products while working in the pharmacy. Many said that because they worked for large companies and business owners, it was part of their role to promote certain products, both medicinal and herbal, which were stocked in the pharmacy:

*'The big companies that employ pharmacists they just want you to sell. I don't think that’s right.'* (Juliet, 45 years’ experience, chain)

*'Community pharmacy, apart from the professional aspect, is also run as a business as well, but as long as it’s an ethical business it’s fine. So basically, you’re just pushing something that you would be selling anyway, but where you’re making a bit more money on it.'* (Stephen, 30 years’ experience, independent)

Some pharmacists perceived that they were trusted less than doctors because they were trying to sell people products and/or medicines:

*'Another thing is the reason why they trust their doctors is because the doctors aren't trying to sell them something. You see, if they come to us and we try and sell them a cough syrup or some cough sweets and things like that, then it’s just about us trying to make some money. That’s what they think anyway.'* (Jane, 3 years’ experience, chain)

However, commercial pressures could be harnessed in a positive way through incentives for pharmacists to help reduce antibiotic prescribing:

*'Sales would be one motivator, two would be probably a public health* [campaign] *where we are incentivised. Like, if training of this actually led to less antibiotics being prescribed, great, that would be something, but that incentivises me as well because I'll be dishing out less antibiotics.'* (Harry, 16 years of experience, chain)

#### Patients’ concerns about the costs of medication

A few pharmacists mentioned that patients may not go to the pharmacist if they are worried about the costs of medication:

*'Patients are still going in to say, "Oh, I've hurt my elbow," and instead of going to pay 16 p in Tesco or whatever* [for pain relief medication]*, they will go to the doctor still which is utterly bizarre. I think it’s not just cost. It might be perception of the cost as well; that it’s going to be expensive; it’s going to be horrible and pricy.'* (James, 20 years’ experience, independent)

### Theme 3: Pharmacist–patient communication

The communication between pharmacists and patients during consultations is crucial. Pharmacists build up long-term relationships with their patients, reinforcing a sense of a neighbourhood pharmacy. They sometimes experienced challenges with language barriers with patients from ethnic minority communities, when managing patients’ expectations (especially around antibiotic prescribing), and patients’ trust in pharmacists.

#### A neighbourhood pharmacy

Good communication with pharmacists played a crucial role in creating a sense of belonging and comfort for patients, making them feel like the pharmacy was an integral part of their neighbourhood:

*'"Hey, do you remember me? I remember when your child was a child, and now they're an adult." That kind of client, you know. It helps … What you used to have 20, 30 years ago where you would walk in and you would see your local GP, and go, "Hello,* [Name of doctor], *how’s it going? I'm going to go see* [Name of pharmacist] *down the road and get my meds."'* (Tom, 16 years’ experience, chain)

#### Language barriers

Pharmacists encountered linguistic challenges when engaging in communication with people who do not speak English confidently. These challenges were evident when pharmacists provided guidance on how to use antibiotics and herbal remedies for self-management. There were concerns around what information was understood by the patient and the reliance on other staff to translate:

*'Yes, language barrier. Where I work, it’s an Asian community, majority, and a lot of them don't speak English well. So, trying to explain that they don't need to go to the hospital is difficult a lot of the time. Luckily, I do have a pre-reg who can speak different languages, so it is a bit of a help, but sometimes, it’s borderline impossible with trying to get that message across.'* (Paula, 3 years’ experience, chain)

#### Managing patients’ expectations

Some pharmacists discussed the challenges of managing expectations from patients who insist on antibiotics because they can get them OTC in their own country:

*'Again, it’s the prescribing habits that other countries have, because obviously there’s quite a few countries that a lot of antibiotics are available over the counter and they're so widely used that most people think nothing’s going to do it unless they have a course of antibiotics and it’s just what they're used to.'* (Jane, 3 years’ experience, chain)

Pharmacists spoke about challenges in dealing with patients who had received antibiotics from their GPs (mostly for UTIs) and then subsequently expected to get them from the pharmacist:

*'A lot of times when patients come into my practice, they go, "The doctor gave me antibiotics last time, why can't you give it to me now?"... I think it makes it very tough for me when I'm presented with something and obviously, the patient’s expectations as well might be a lot different, they might want the antibiotic because they need to get well.'* (Harry, 16 years’ experience, chain)

## Discussion

### Summary

Pharmacists recognised their key role in providing self-management advice for acute conditions. While herbal medicines were seen as a potential alternative approach to symptom management, some pharmacists generally felt ill-equipped to be offering advice, either because of their lack of knowledge around herbal medicines or the perceived lack of evidence of benefit. Pharmacists identified a training need to be filled before they would have the confidence to incorporate this into clinical practice.

Pharmacists also spoke about the tension between commercial drivers to sell products, and patients looking for cost-effective solutions and pharmacists wishing to provide evidence-based solutions. Linked to this was the perception that patients may be suspicious of pharmacists’ motives for recommending products that required a payment. Indeed, pharmacists reported pressure to sell, regardless of whether they worked in independent pharmacies or in chain stores. Participants also highlighted the language factor and different regulatory environments that may result in a higher demand for antibiotics.

### Strengths and limitations

This qualitative study has provided insights into the role of community pharmacists in advising herbal medicine as part of self-management for acute infections. The study involved a diverse range of various ethnic backgrounds and pharmacy types, which enriched the collected data with diverse perspectives, focusing explicitly on herbal medicine within self-management.

The sample size and the routes for recruitment were limited, and the pharmacists who participated may have been more engaged with the research topic than others. They worked in pharmacies from a variety of areas but the median Index of Multiple Deprivation was 7 (on a scale of 1–10, where 1 is the most deprived and 10 is the least deprived). These pharmacists may not be representative of those from more deprived areas. Their experiences and challenges may vary widely according to patients’ demographics, especially their culture, attitudes towards herbal medicines and antibiotics, and ability to pay for herbal and other OTC remedies. There were also limited data to understand the extent to which pharmacy technicians prescribe medicines under PGDs.

### Comparison with existing literature

It is a priority to provide healthcare professionals with effective messages to ensure they can continue to meet the needs and expectations of their patients.^26^ Despite a succession of public health messages regarding antibiotic overuse, public awareness remains suboptimal,^27^ and pharmacists could play an increased role in antimicrobial stewardship^28,29^ and safeguarding people from inappropriate OTC medications.^18^ However, critics argue that expanding the prescribing authority of pharmacists could compromise patient safety, particularly if pharmacists are not adequately trained or supported in their new role.^30^

Future campaigns could emphasise positive action to relieve symptoms, rather than solely emphasising the negative message to avoid antibiotics. Previous antibiotic users and certain groups, such as those from cultures with higher antibiotic use, may require specific information tailored to their needs. To address these needs, an information leaflet on self-care has been developed with the input of patient representatives, which can be used in community pharmacies to provide guidance and advice to patients seeking to manage their own healthcare needs.^31^

Previous studies have highlighted some constraints in community pharmacies, including time constraints, inadequate knowledge among unskilled staff, space limitations,^32,33^ and subpar knowledge of dietary supplements, as revealed by a 64% median knowledge score in a systematic review.^34^ A qualitative study in the UK suggested a disparity between pharmacists’ theoretical understanding of self-care and their actual representation of roles in supporting self-care.^17^ Barriers to offering self-care support include priority given to dispensing, the pharmacy contract structure, lack of incentives, and patients' expectations and lack of awareness of community pharmacies' roles.^17^ Addressing these challenges may require a multifaceted education intervention, including targeted antibiotic advice and feedback for patients and pharmacy staff.^33^ Similar issues in other countries underscore the need to develop training and guidelines for pharmacists on providing self-care advice, including information on effectiveness and safety of common herbal products and supplements available under the THR list in the UK.^19,35^

### Implications for research and practice

As the policy shifts to move assessment of ARI into the community, assessment and self-management advice is likely to become more prevalent in the pharmacy setting, therefore ensuring pharmacists are well equipped for non-antibiotic advice is a high priority.^36^ Future research is needed to explore effective approaches for raising public awareness about self-care and reducing patient expectations for antibiotics in the management of common infections. A simulated patient approach may be appropriate to understand how pharmacists interact with patients regarding advice on infections.^37^ To strengthen antibiotic stewardship programmes, it is important to explore the use of incentives and equip community pharmacists with the best available evidence and training on self-management, including the use of herbal medicines. Maximising implementation of evidence-based guidelines on self-management will require minimising costs for patients while maximising profitability for pharmacists of the best non-antibiotic treatments.

Three themes were identified from the data (Figure 1): self-management recommendations; factors influencing pharmacists’ recommendations for acute infections; and pharmacist–patient communication. In conclusion, community pharmacists play a central and increasing role in the management of self-limiting illness. Some pharmacists currently feel ill-equipped to provide best advice on self-management strategies including the use of herbal medicines. In order to maximise the benefits of switching consultations from GPs to community pharmacies, pharmacists need to not only assess and manage acute illness, but also may benefit from having an effective toolkit of management strategies that do not involve antibiotics. Where evidence exists to support use of non-antibiotic medicines for symptom relief, these medicines should be available in all pharmacies.
